# Optimizing Thermomechanical Processing of Bimetallic Laminates

**DOI:** 10.3390/ma16093480

**Published:** 2023-04-30

**Authors:** Radim Kocich

**Affiliations:** Faculty of Materials Science and Technology, VŠB–Technical University of Ostrava, 17. Listopadu 2172/15, 70800 Ostrava-Poruba, Czech Republic; radim.kocich@vsb.cz; Tel.: +420-596-994-455

**Keywords:** bimetallic laminate, rotary swaging, microstructure, electric conductivity, microhardness

## Abstract

Thermomechanical processing combining plastic deformation and heat treatment is a favorable way to enhance the performance and lifetime of bimetallic laminates, especially those consisting of metals, which tend to form intermetallic layers on the interfaces when produced using methods involving increased temperatures. The presented work focuses on optimizing the conditions of thermomechanical treatment for an Al + Cu bimetallic laminate of innovative design involving a shear-strain-based deformation procedure (rotary swaging) and post-process heat treatment in order to acquire microstructures providing advantageous characteristics during the transfer of direct and alternate electric currents. The specific electric resistivity, as well as microhardness, was particularly affected by the structural features, e.g., grain size, the types of grain boundaries, and grain orientations, which were closely related to the applied thermomechanical procedure. The microhardness increased considerably after swaging (up to 116 HV02 for the Cu components), but it decreased after the subsequent heat treatment at 350 °C. Nevertheless, the heat-treated laminates still featured increased mechanical properties. The measured electric characteristics for DC transfer were the most favorable for the heat-treated 15 mm bimetallic laminate featuring the lowest measured specific electric resistivity of 22.70 × 10^−9^ Ωm, while the 10 mm bimetallic laminates exhibited advantageous behavior during AC transfer due to a very low power loss coefficient of 1.001.

## 1. Introduction

Processes of plastic deformation are advantageous not only for imparting the required geometries to the produced work pieces and components but also for controlling their structures and, consequently, their properties, lifetime, and performance [1,2,3]. The methods can be divided into two basic groups, i.e., conventional (e.g., rolling, forging, drawing, and extrusion) and unconventional methods. Among the favorable unconventional methods are the methods of intensive and severe plastic deformation (IPD and SPD methods). Such processes are based on introducing (high amounts of) shear strain into the processed work pieces, by which they induce the fragmentation of the grains and the development of the substructure [4,5]. The repeated occurrence of grain fragmentation and subgrain nucleation and growth finally leads to the improvement of the properties, especially the strengthening, of the processed material [6,7]. In other words, strengthening by dislocations, i.e., the presence of low-angle grain boundaries (LAGBs), and Hall–Petch strengthening, i.e., the presence of high-angle grain boundaries (HAGBs), are the key factors contributing to the overall flow stress of a material [8]. Nevertheless, their ratio depends significantly on the amount of imposed strain and related structure phenomena [9].

Equal-channel angular pressing (ECAP) [10,11] and other ECAP-related methods (e.g., equal-channel angular pressing with partial back pressure (ECAP-PBP) [12], twist-channel angular pressing (TCAP) [13], twist-channel multi-angular pressing (TCMAP) [14], etc.), twist extrusion (TE) [15], accumulative roll bonding (ARB) [16], high-pressure torsion (HPT) [17], and friction stir processing and welding (FSP and FSW) [18,19] are among the known SPD processes used by researchers worldwide to favorably influence the structures of various processed metallic materials to achieve ultra-fine-grained (UFG) structures. However, although they are favorable for the mentioned grain refinement and enhancement of mechanical properties, SPD methods feature typical disadvantages; mostly, the processed material volume is limited by the applied die and available power output of the machine [20,21].

The IPD method of rotary swaging is an industrially applicable technology, which can advantageously be used to produce long (axisymmetric) products, including electro-conductive wires [22]. Moreover, according to the geometry of the dies, the method can be used to fabricate products of complex shapes and geometries [23]. Due to small increases in compressive strain, which are induced by the impacts of a set of dies rotating at a high speed around the processed work piece, the homogeneity of the (residual) stress across the work piece is generally improved, which enables the method to also be used for the processing of challenging materials (powder-based materials, composites, laminates, etc. [24,25,26]). Similar to SPD methods, swaging generally induces grain refinement, the formation of the (sub)structure, and the development of dynamic restoration processes, the mutual combination of which consequently leads to enhanced properties of the swaged products [27,28,29]. In other words, the method generally improves the mechanical properties of processed work pieces without deteriorating their lifetime or performance. However, its overall effect depends on several factors, primarily the processing parameters and the type of swaged material [30,31,32]. Among the main influencing factors is also the reduction ratio, which affects the depth of the penetration of the imposed strain and, thus, the distribution and homogeneity of the imposed strain across the cross-section of the work piece. As the dies primarily affect the periphery of the processed work piece, the imposed strain is the greatest in its peripheral region and decreases gradually towards its axial region [33]. Nevertheless, this strain distribution inhomogeneity generally decreases with an increasing reduction ratio [34].

Bimetallic laminates consist of (at least) two different metals or alloys. Their combination usually results in advantageous properties of the final product [35,36]. In other words, the properties of the laminated product are more advantageous (e.g., for a specific application) than those of the individual metallic components. Due to its wide range of applicability, the system of Al + Cu is very popular. Among the possible applications are, e.g., Al + Cu clad armored cables [37], laminated tubes for air conditioners [38], and rail transit wires [39]. In particular, the application of Al + Cu bimetallic laminates for electroconductive applications is highly promising, since the (partial) replacement of Cu by Al results in decreased weight and enhanced mechanical and utility properties of the electroconductive cable with no significant deterioration of the electric characteristics [40].

Before designing a bimetallic laminate and its production procedure, its intended application should be considered, as the selected component metals and designed volume ratio and stacking sequence influence the final properties of the laminate. The production methods using elevated/high temperatures (e.g., cladding or welding [41,42,43,44,45]) are not very advantageous since they introduce temperature inhomogeneity, which can deteriorate the structures (e.g., by introducing precipitation, the formation of intermetallics, or undesirable grain growth) and, consequently, the mechanical properties of the bimetallic laminate (despite the fact that they provide a sufficient bonding strength) [46]. Therefore, the production of Al + Cu bimetallic laminates under cold conditions, i.e., via intensive/severe deformation processing, is promising [47,48]. The shear-strain-based methods of intensive/severe plastic deformation provide sufficient energy for the individual metals to develop a high-quality bond at mutual interfaces, even under cold conditions, which also advantageously suppresses the development of undesirable intermetallic phases.

The presented work focuses on the processing of an Al + Cu bimetallic laminate of our own design via thermomechanical treatment with the implementation of room-temperature rotary swaging and post-process heat treatment at 350 °C. The main aim of the study is to evaluate the effects of processing conditions on the structure development and related mechanical and electric characteristics of the laminate. Special attention is given to the evaluation of the effects of the applied swaging degree and the possible combination with post-process heat treatment.

## 2. Materials and Methods

### 2.1. Experimental Material

**Figure 1:**
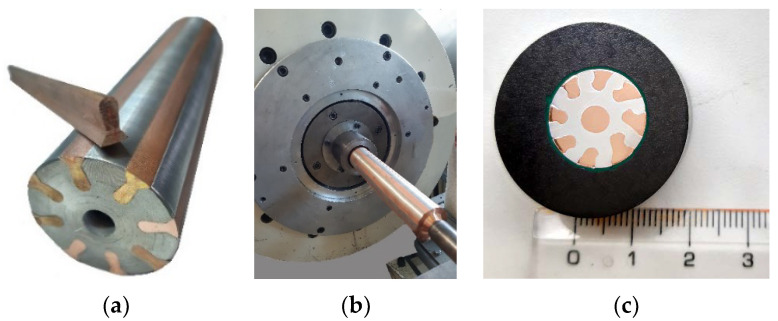
Assembling of the billets (**a**); ready to swage (**b**). Cross-sectional cut through the 15 mm swaged bimetallic laminate (**c**).

The original materials, which were used to manufacture the bimetallic laminates, were commercially pure electro-conductive copper, i.e., CP Cu, and commercially pure electro-conductive aluminum, i.e., CP Al. The impurities within the metals were 0.016% P, 0.002% Zn, and 0.002% O for the CP Cu and 0.24% Fe, 0.21% Si, and 0.05% Cu for the CP Al. The design of the Al + Cu laminate ensued from the experience acquired during previous research on different types of Al + Cu laminates (e.g., [22,49]). To be specific, based on previously acquired experience, the CP Cu was primarily involved at the periphery of the laminate; i.e., in the lamellas, which were arranged in a stellulate pattern; and in its axis. (Figure 1a) Placing the Cu primarily at the periphery of the laminate is also advantageous for the prospective usage of the bimetallic laminate for the transfer of the alternating current (AC), which is known for its inhomogeneous distribution across the cross-sections of conductors. In other words, the current density across the cross-section of a conductor during AC transfer is inhomogeneous due to the occurrence of the skin effect [50,51]. The CP Al then formed the matrix, i.e., the major bulk volume of the laminate. The original diameter of the composite billets was 50 mm. During processing, these billets were gradually swaged at room temperature down to the diameter of 15 mm and, finally, to the diameter of 10 mm (see Figure 1b for the billet ready to be swaged, and Figure 1c for a cross-sectional cut through the 15 mm swaged bimetallic laminate). These two final swaging passes were selected for the subsequent investigations leading to the optimization of the swaging degree based on the assessment of the (sub)structure and the related mechanical and electrical properties. The swaging degrees after the investigated swaging passes were calculated using relation (1):

(1) $$\varphi =\ln\left(\frac{S_{0}}{S_{n}}\right)$$

where *S*_0_ and *S_n_* are the cross-sectional areas of the laminate at the input and output of the swaging dies, respectively. The calculated values of the swaging degrees were 2.4 for the 15 mm laminate and 3.2 for the 10 mm laminate.

In order to find out the effects of temperature on the structures, both the 15 mm and 10 mm swaged laminates were subjected to a post-process heat treatment consisting of a 15 min dwell in a furnace pre-heated to the temperature of 350 °C and cooling in air. During the previous experiments, we found that annealing treatments applied after rotary swaging tend to stabilize the structures of Al + Cu laminated composites via structure restoration. However, the used temperature should be lower than 350 °C, as temperatures above this critical value tend to promote the undesirable formation of brittle intermetallics at the interfaces, as well as possibly secondary recrystallization, i.e., the growth of grains [49].

### 2.2. Structure Observations

Structure analyses were performed using scanning electron microscopy (SEM—a Tescan Lyra 3 XMU FEG/SEMxFIB device with a Symmetry EBSD detector, Tescan Orsay Holding a.s., Brno, Czech Republic). The samples for the electron backscatter diffraction (EBSD) analyses, acquired by transversally cutting the processed laminates, were prepared via manual grinding, manual polishing using alcohol-based diamond solutions (Struers GmbH, Roztoky u Prahy, Czech Republic), and final electrolytic polishing. During the scanning of the 250 × 150 µm^2^ areas used for further evaluations, the applied scan step was 0.25 µm. The analyses were evaluated using Aztec Crystal software (Oxford Instruments, Abingdon, UK). The considered limiting values during the evaluations of the grain boundaries were 15° for HAGB and 5° for LAGB. Texture analyses were performed with the maximum deviation from the ideal orientation of 15°. Detailed analyses of the substructures of the Cu lamellas were performed using a JEM-2100 transmission electron microscope (TEM, JEOL, Tokyo, Japan) operating at 200 kV. The samples for the TEM observations were prepared using the focused ion beam (FIB) technique assembled on the above-mentioned Tescan Lyra 3 XMU SEM device. The preparation of the TEM samples involved the gradual milling of the particularly thin lamella with Ga ions to a final thickness of approx. 120 nm.

### 2.3. Evaluation of Properties

The measurements of the HV02 Vickers microhardness were performed on perpendicular cross-sectional cuts of the swaged and heat-treated bimetallic laminates. The used equipment was a Zwick/Roell device (Zwick Roell CZ s.r.o., Brno, Czech Republic). The average microhardness values were calculated for each of the bimetallic laminates from ten individual indents randomly performed across the cut cross-sections. The load for each individual indent was 200 g, and the load time dwell was 10 s.

To assess the electric characteristics of the swaged and heat-treated bimetallic laminates, as well as the conductors prepared from the CP Cu and CP Al metals, during the transfer of direct and alternate currents (DC and AC), a SIGMATEST 2.070 measuring device (FOERSTER TECOM s.r.o, Prague, Czech Republic) was used. This device is a high-tech eddy current portable piece of equipment that enables one to measure the electric resistivity, and thus to assess the electric conductivity, of non-ferromagnetic metallic materials using a measuring probe. This equipment can also be advantageously used to determine the electric characteristics of work pieces with small dimensions [52]. At first, we performed the calibration of the device with the help of two default specimens with different (known) electric conductivities. Following the calibration, the probe was ready to be used for measuring the desired electric characteristics of the processed conductors and bimetallic laminates.

## 3. Results and Discussion

### 3.1. Analyses of Grains

The results of the grain size evaluations are summarized in Table 1. The grain size was assessed via the maximum Feret diameter. This parameter is characterized as the maximum distance between two individual points defining a single grain. As can be seen, the swaging evidently induced grain refinement in both the original metals. The investigated characteristic dimensions were larger for both the Cu and Al components of the 15 mm swaged bimetallic laminate than for the components of the 10 mm one with regard to the average max. Feret diameter, the minimum and maximum grain size, and the standard deviation from the average grain size value. Comparing the average grain sizes of the 15 mm and 10 mm bimetallic laminates with those of the original metals, the structures of the Cu components were refined. However, the grain refinement within the Al was more significant than that observed within the Cu primarily due to the fact that Al is highly susceptible to the imposed shear strain (primarily due to its intrinsic properties, such as the stacking fault energy), which facilitates the plastic flow of the Al matrix [53]. Moreover, the observed difference between the grain size values of the Al matrices of the 15 mm and 10 mm bimetallic laminates was negligible. This phenomenon points to the fact that the grain refining ability of the shear strain imposed by the rotary swaging to the Al structure reached its maximum level at the applied swaging degree of 2.4. In other words, the rates of dynamic strengthening and restoration within the Al matrix of the bimetallic laminate swaged to the diameter of 15 mm became equalized [54]. Dalla Torre et al. [55] presented similar conclusions, according to which deformation via shear strain imposed at room temperature does not result in further observable changes in the morphology of the grains at the point where dislocation clusters and stacking faults are randomly scattered in the interiors of dynamically restored grains, which become equiaxed as the shear strain imposed during a continuing swaging process gradually increases.

After the heat treatment, the average grain sizes within the Al matrices were comparable to those acquired for the swaged laminates. In other words, the average grain size value remained identical for the 15 mm bimetallic laminate, and it increased negligibly for the 10 mm one. However, the minimum observed grain size increased and the maximum grain size decreased for both the diameters when compared to the swaged laminates. Consequently, the standard deviations for the grains within the Al matrices decreased for both the diameters of the bimetallic laminate, and, thus, the homogeneity of the structures of the Al matrices, from the viewpoint of grain size, increased after the heat treatment. The structure of the Al within both the swaged laminates can thus be considered as more or less stable since the temperature of 350 °C did not provoke any substantial grain growth or structure changes [54]. Nevertheless, the effect of the heat treatment on the Cu components of the bimetallic laminates was not so unambiguous.

The Cu components of the 15 mm heat-treated bimetallic laminate exhibited a simultaneous decrease in the average grain size and standard deviation (similar to the Al matrix). However, the grains within the Cu components of the 10 mm bimetallic laminate exhibited slight growth after the heat treatment, and the standard deviation for this laminate also increased. This structure behavior supports a hypothesis stating that the energy introduced to the 15 mm bimetallic laminate by the heat treatment brought about enough energy for the Cu structure to undergo restoration followed by secondary recrystallization, i.e., (undesirable) grain growth. However, the 10 mm bimetallic laminate, which was subjected to a higher swaging degree during processing, featured a higher level of accumulated energy after swaging. Therefore, the energy provided by the heat treatment primarily induced visible grain growth within this laminate. The hypothesis is supported not only by the results discussed below of the (sub)structure analyses but also by the results acquired within previously performed experimental works on Al + Cu laminates of different cladding designs (e.g., [49]), in which the effects of different combinations of imposed strain + temperature were studied and discussed in detail.

### 3.2. Structure Analyses with Focus on Cu

**Figure 2:**
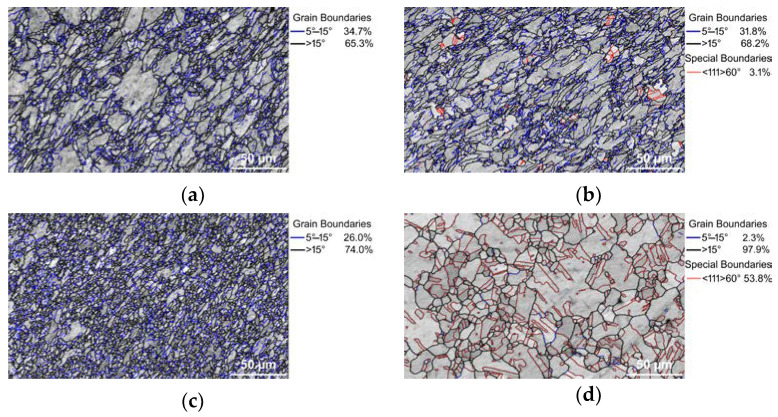
Band contrast EBSD images with depicted grain boundaries within Cu lamellas of bimetallic laminates: 15 mm RS (**a**); 15 mm HT (**b**); 10 mm RS (**c**); 10 mm HT (**d**).

**Figure 3:**
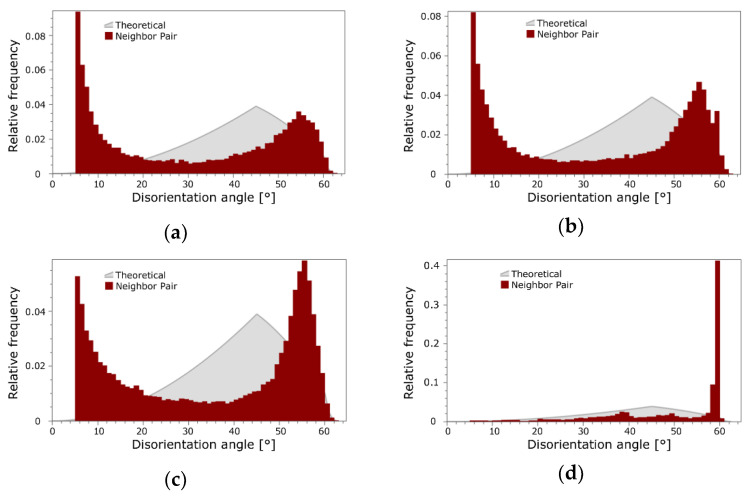
Disorientation angle distributions for Cu lamellas of bimetallic laminates: 15 mm RS (**a**); 15 mm HT (**b**); 10 mm RS (**c**); 10 mm HT (**d**).

As the Cu components are supposed to primarily affect the electric conductivity, detailed (sub)structure analyses are focused on the Cu structures. The above-drawn hypotheses were confirmed by grain boundary analyses. Figure 2a,b show the band contrast images of the structures of the 15 mm rotary-swaged (RS) bimetallic laminates, while the band contrast images of the structures of the 15 mm swaged and heat-treated (HT) bimetallic laminates are shown in Figure 2c,d. The disorientation angle distribution charts for the samples are depicted in Figure 3a,d. As documented by the mentioned figures, the heat treatment did not provoke any substantial changes in the LAGB vs. HAGB ratio within the structure of the Cu lamellas of the bimetallic laminate swaged to 15 mm, although the heat-treated structure featured the local development of twins (Figure 2b). In other words, the LAGB vs. HAGB ratio for the Cu structures of both the processed 15 mm laminates was approx. 1/3 to 2/3. Most probably, recrystallization occurred locally within the Cu structure during the heat treatment, but the time period needed for its full development was longer than the applied time dwell [54]. This supposition is also supported by the above-mentioned grain size observations and the disorientation frequencies in Figure 3a,b. However, the structure within the Cu lamellas of the 10 mm bimetallic laminate exhibited evident grain growth and a change in the LAGB vs. HAGB ratio after the heat treatment. The treatment thus promoted recrystallization, as well as the massive development of annealing twins (compare Figure 2c,d and Figure 3c,d). The fraction of HAGB within the Cu structure of the 10 mm laminate increased from 74.0% to 97.9% after the heat treatment; 53.8% of the HAGBs were <111>60° twin boundaries. This fact is also evident in Figure 3d showing that the majority of the grain boundaries within the structure of the Cu lamellas of the heat-treated 10 mm bimetallic laminate featured a disorientation angle of 60°. This phenomenon can be attributed to the fact that the tendency of the structure of a material featuring an FCC lattice to perform structure changes via twinning increases with increasing flow stress, which can be promoted, e.g., by increasing the ambient temperature via the application of a heat treatment [56]. This tendency even increases for materials, the grains within which are refined by intensive deformation to the ultra-fine or nano-scales prior to the heat treatment [57]. Corresponding to this finding, the Cu structures of the herein presented 10 mm bimetallic laminates exhibited a stronger tendency to form twins than those within the 15 mm laminates.

### 3.3. Texture

**Figure 4:**
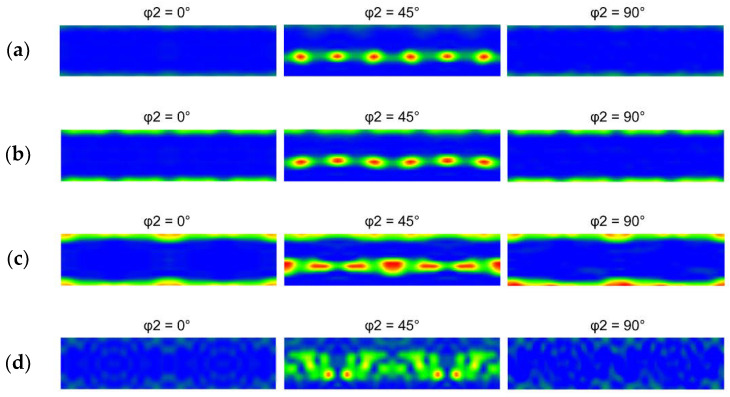
ODF representation of grain orientations for structures of Cu bimetallic lamellas of laminates: 15 mm RS (**a**); 15 mm HT (**b**); 10 mm RS (**c**); 10 mm HT (**d**).

**Figure 5:**
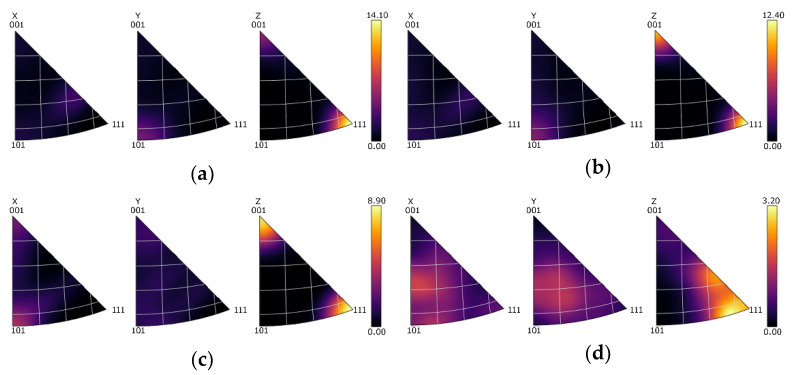
IPF texture images of structures of Cu bimetallic lamellas of laminates: 15 mm RS (**a**); 15 mm HT (**b**); 10 mm RS (**c**); 10 mm HT (**d**).

An optimized deformation texture can significantly affect the electric conductivity in a positive manner. Figure 4a–d show the orientations of the grains within the Cu lamellas of the 15 mm RS, 15 mm HT, 10 mm RS, and 10 mm HT bimetallic laminates, respectively, using the Euler angles via Orientation Distribution Functions (ODFs), which are plotted in the following ranges of the characteristic angles: 0 < *φ*_1_ > 360°, 0 < *ϕ* > 90°, and *φ*_2_ are sections of 0°, 45°, and 90°. In order to quantify the texture intensity within the individual laminates, inverse pole figures (IPFs) for the 15 mm RS, 15 mm HT, 10 mm RS, and 10 mm HT bimetallic samples are depicted in Figure 5a–d.

**Figure 6:**
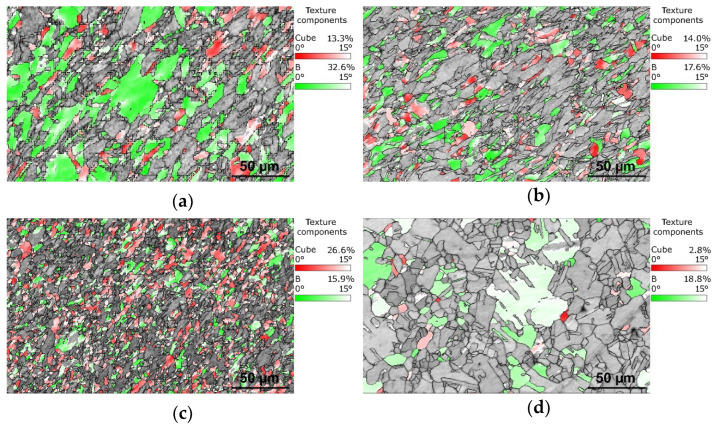
Texture components by Euler angles within structures of Cu lamellas of bimetallic laminates: 15 mm RS (**a**); 15 mm HT (**b**); 10 mm RS (**c**); 10 mm HT (**d**).

The 15 mm swaged bimetallic laminate exhibited a very strong texture. The ideal texture orientations featuring the highest intensity belonged to the *B* shear texture fiber defined with Euler angles of *φ*_1_ = 90°, *ϕ* = 55°, and *φ*_2_ = 45° (Figure 4a). The fiber very well corresponded with the (111)||SD (swaging direction) fiber (see also Figure 5a). In other words, the Cu grains within the 15 mm swaged bimetallic laminate exhibited preferential texture orientations typical for FCC metals subjected to severe shear strain [22]. As evident in Figure 4b and Figure 5b, the heat treatment decreased the maximum texture intensity observed for the 15 mm laminate. The *B* texture fiber was still evident within the heat-treated structure. However, a portion of the grains recrystallized, and the observed intensity of the ideal *B* shear deformation texture fiber was comparable to the intensity of the *Cube* ideal recrystallization texture (Euler angles of *φ*_1_ = 0°, *ϕ* = 0°, and *φ*_2_ = 0–90°); note the increase in the intensity of the (001)||SD fiber evident in Figure 5b. These findings were confirmed by the detailed analyses of the orientations of the individual grains within the Cu structures of the 15 mm bimetallic laminates depicted in Figure 6a,b. These figures clearly show that the intensity of the *B* deformation texture fiber dominated within the swaged structure, whereas after the heat treatment, its intensity decreased and became comparable to that of the *Cube* ideal recrystallization texture orientation (the intensity of which slightly increased after the heat treatment).

The maximum intensity of the texture observed within the 10 mm swaged bimetallic laminate (see Figure 4c,d and Figure 5c,d) was lower than that within the 15 mm swaged one, as well as lower than that within the 15 mm laminate after the heat treatment. The dynamic recrystallization occurring during swaging down to the final laminate diameter of 10 mm thus promoted texture randomization, which went hand in hand with the above characterized grain refinement and increase in HAGB fraction. The structure still featured a relatively strong *B* shear texture fiber, which is also evident in Figure 6c showing the texture orientations in the individual Cu grains of the 10 mm bimetallic laminate. However, the intensity of the *Cube* recrystallization texture within this structure was very high, and it almost doubled when compared to both the Cu structures of the 15 mm bimetallic laminates (swaged and heat-treated). From the viewpoint of the recrystallized fraction, one can conclude that swaging to 15 mm combined with the post-process heat treatment at 350 °C was not as efficient for the promotion of recrystallization within the Cu structure at the higher reduction ratio, i.e., direct swaging down to 10 mm.

The heat treatment applied to the 10 mm bimetallic laminate then introduced notable texture changes. The maximum texture intensity decreased, as can be seen in Figure 4d and Figure 5d, but the structure still featured ideal texture orientations of a high intensity—the intensity of the *B* texture fiber increased after the heat treatment, but the intensity of the *Cube* recrystallization texture decreased to a very low level (compare Figure 6c,d). Such structure development can be explained as follows: the intensive shear strain imposed during the swaging process promoted repeated grain fragmentation, followed by the development of the substructure and the nucleation of new grains [53]. Therefore, the structure within the bimetallic laminate subjected to a higher swaging degree, i.e., swaged to 10 mm, underwent a greater number of repetitions of the grain nucleation/fragmentation processes during the deformation processing than the one swaged with a lower swaging degree, i.e., to 15 mm. The high intensity of the *Cube* texture orientation within the swaged structure of the 10 mm bimetallic laminate (Figure 6c) corresponded to a stage in which (partial) dynamic recrystallization barely occurred. Because of this, the subsequent heat treatment did not promote recrystallization, as observed for the 15 mm bimetallic laminate, but only structure restoration via the recovery and growth of a portion of the deformed grains, via the effect of which the fraction of the grains featuring the *B* fiber ideal orientations increased slightly after the heat treatment (Figure 6d).

### 3.4. Substructure

**Figure 7:**
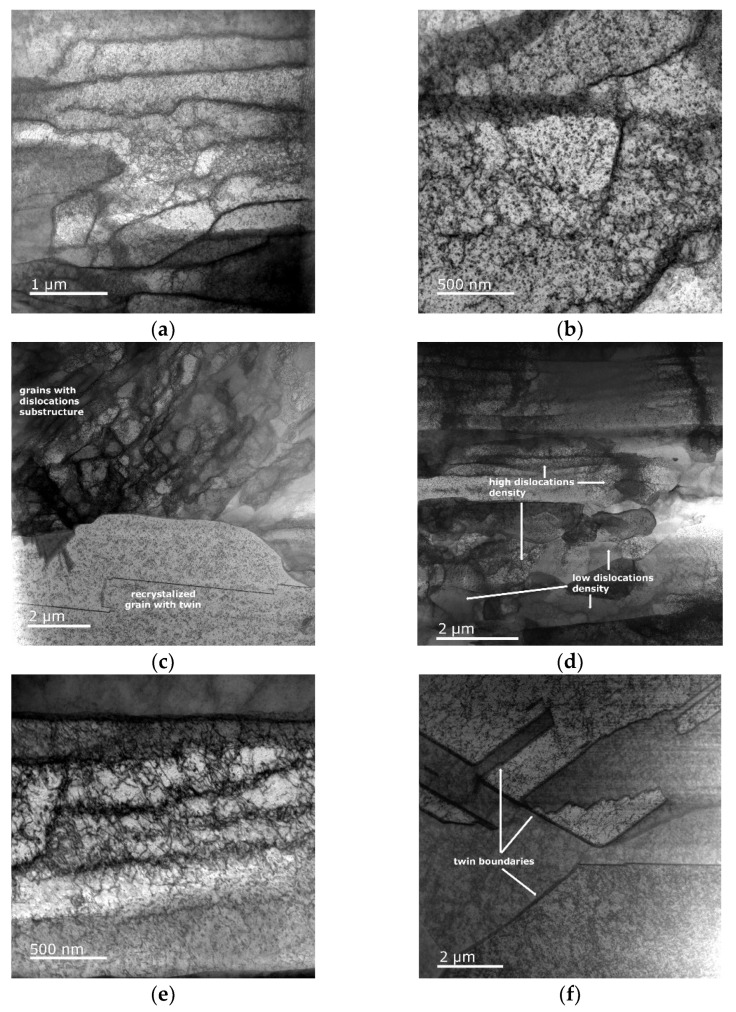
TEM substructure images of Cu lamellas of bimetallic laminates: 15 mm RS (**a**,**b**); 15 mm HT (**c**); 10 mm RS (**d**,**e**); 10 mm HT (**f**).

In order to confirm the above-drawn conclusions on structure recrystallization and recovery, the development of the substructures within the Cu lamellas of the swaged and heat-treated bimetallic laminates was observed in detail via scanning TEM (i.e., STEM). As evident in Figure 7a,b, swaging the bimetallic laminate to the diameter of 15 mm resulted in a substantial accumulation of lattice defects and the formation of the substructure. In other words, the Cu grains featured a high density of accumulated dislocations. The heat treatment then induced the partial recrystallization of the structure and the related annihilation of dislocations as the mobility of the dislocations increased due to the thermally activated dislocation motion [58]. Figure 7c evidently shows the heterogeneous structure and the recrystallized dislocation-free grains, which were mixed with (still) highly deformed grains featuring a developed substructure. This finding confirms the above discussed conclusions based on the results of the texture analyses (e.g., Figure 6b). The STEM image in Figure 7c also depicts a twin within a recrystallized grain, which confirms the development of twin boundaries within the Cu structure of the 15 mm bimetallic laminate during heat treatment (Figure 2b). Figure 7d–f show the STEM images of the Cu substructure acquired from the 10 mm bimetallic laminate. The simultaneous presence of recrystallized and deformed grains, as discussed above, can be seen in Figure 7d. In other words, the Cu structure of this swaged laminate also exhibited the presence of grains featuring a high dislocation density (e.g., Figure 7e) but also (recently) recrystallized grains with a lesser occurrence of dislocations. After the heat treatment, the 10 mm bimetallic laminate exhibited dislocation annihilation and the massive development of twins (as also documented by the grain boundary analyses), which is confirmed by the STEM image depicted in Figure 7f.

### 3.5. Mechanical Properties

**Figure 8:**
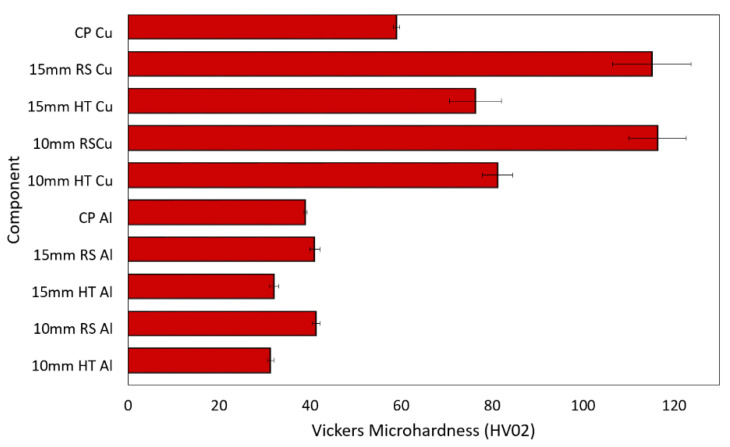
Vickers microhardness for original metals and components of bimetallic laminates.

The HV02 Vickers microhardness was evaluated for the 15 mm and 10 mm swaged and heat-treated bimetallic laminates, as well as for the conductors swaged from the original CP Cu and CP Al metals. The results are summarized in Figure 8. The average microhardness values for the Al and Cu components of the 15 mm and 10 mm swaged bimetallic laminates were more or less comparable (compare 115.2 HV02 for the Cu of the 15 mm laminate and 116.9 HV02 for the Cu of the 10 mm laminate, and compare 41.0 HV02 for the Al of the 15 mm laminate and 41.2 HV02 for the Al of the 10 mm laminate). The comparison of the measured values of microhardness with the values acquired for the conductors swaged from the original Al and Cu metals points to deformation hardening induced by swaging. Considering the different imposed swaging degrees, the observed increases in the HV02 values (especially for the Cu components) were related to the (different) phenomena occurring within the structures of the 15 mm and 10 mm bimetallic laminates. The grains within the Cu of the 15 mm swaged bimetallic laminate were not as refined as those within the 10 mm swaged one. Nevertheless, the grains featured substantial substructure development, i.e., a high dislocation density, which was, most probably, the primary feature contributing to the high microhardness of this laminate. However, the substructure within the Cu grains of the 10 mm swaged bimetallic laminate was not as developed, but the grains were more refined; i.e., the average grain size was smaller, which resulted in a higher volume fraction of grain boundaries within the structure. Grain boundaries are generally known to feature a higher energy than the interiors of grains, and they also typically act as obstacles for dislocation movement, by which they contribute to the strengthening of metallic materials [53]. Therefore, the primary factor contributing to the high microhardness of the 10 mm swaged bimetallic laminate was the small grain size.

The post-process heat treatment introduced structure softening for both the investigated swaged bimetallic laminates; however, the decrease in microhardness induced by the heat treatment was more substantial for the 15 mm laminate. In other words, the Cu components within the 10 mm heat-treated laminate featured a higher microhardness than those within the 15 mm one. This phenomenon was related to the observed and above-characterized (sub)structure development—contrary to the 15 mm one, the 10 mm bimetallic laminate exhibited dynamic recrystallization during swaging, and, thus, the subsequent heat treatment was more likely to promote the annihilation of dislocations at the expense of structure softening via recrystallization.

### 3.6. Electric Conductivity

**Figure 9:**
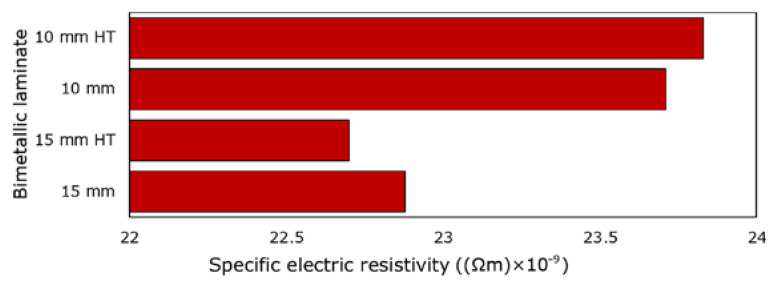
Experimentally measured specific electric resistivity for processed bimetallic laminates.

The measurement of the specific electric resistivity *ρ* was used to experimentally characterize the DC transfer through the 15 mm and 10 mm swaged (and heat-treated) bimetallic laminates. The acquired values are shown in Figure 9 depicting that the electric resistivity of the 10 mm swaged bimetallic laminate was slightly higher than the electric resistivity of the 15 mm swaged one. Although the applied heat treatment affected the structures of the bimetallic laminates substantially, the specific electric resistivity measured after the treatment was comparable to the value acquired for the swaged bimetallic laminate for both the conductor diameters. The highest specific electric resistivity of 23.83 × 10^−9^ Ωm was measured for the 10 mm heat-treated bimetallic laminate, while the lowest electric resistivity of 22.70 × 10^−9^ Ωm was measured for the 15 mm heat-treated bimetallic laminate. In other words, the 15 mm heat-treated laminate featured the highest observed electric conductivity. This can be attributed to several factors; the Cu structure of the 15 mm heat-treated laminate featured restored grains with a decreased density of dislocations (Figure 7c), which act as obstacles for the movement of electrons. Moreover, the laminate featured a favorable texture—the structure exhibited a combination of a recrystallization texture (grains with a restored substructure free of obstacles) and a *B* fiber texture (grains advantageously elongated along the axis of the swaged laminate, i.e., the direction of movement of the electrons). However, the high electric resistivity of the 10 mm heat-treated bimetallic laminate can be attributed to the refined grains (high volume fraction of grain boundaries, i.e., obstacles for the movement of electrons), as well as the massive occurrence of micro-sized twins. The presence of twin boundaries, especially nano-twin ones, within the structure of electroconductive materials was hypothesized by others [59] to possibly improve the electric conductivity, since the occurrence of twin boundaries can result in a decreased density of lattice defects at the regular boundaries. Nevertheless, twinning also subdivides the grain and results in the reorientation of a portion of the crystal lattice [56]; i.e., it impairs the texture, which could have been purposefully tailored to advantageously correspond to the direction of movement of the electrons. Based on these facts, the micro-sized twinning observed within the Cu structure (nano-sized twins were not observed) of the 10 mm heat-treated bimetallic laminate most probably negatively affected the electric conductivity.

Last but not least, an experimental evaluation of the characteristics of AC transfer for both the processed 15 mm and 10 mm bimetallic laminates was performed. For this task, it was necessary to perform measurements and computations of specific parameters, i.e., the specific electric resistivity *ρ* and resistance *R*, for conductors swaged from the original CP Al and CP Cu metals. The values were *R* = 225.1×10^−6^ Ω (Cu) and *R =* 441.3 × 10^−6^ Ω (Al), and ρ = 17.468 × 10^−9^ Ωm (Cu) and *ρ =* 28.772 × 10^−9^ Ωm (Al). The effect of increasing the imposed swaging degree on the power loss during AC transfer, *dP_AC_*, and DC transfer, *dP_DC_*, is evident in Table 2, which shows that the *dP_AC_* power loss was higher than the *dP_DC_* power loss for both the examined diameters of the swaged (and heat-treated) bimetallic laminates.

In order to assess the (different) behaviors of the laminates during DC and AC transfers, coefficient *k_d_*, arising from dividing the *dP_AC_* power loss value by the *dP_DC_* power loss value for each laminate (Table 2), can be computed. The coefficient characterizes the increase in the resistance of a conductor during AC transfer when compared to DC transfer. The final computed values of coefficient *k_d_* for the CP Al and CP Cu, as well as for the swaged (and heat-treated) bimetallic laminates, are also depicted in Table 2. As can be seen, the coefficient was generally higher for the 15 mm conductors and lower for the 10 mm ones. Moreover, it was higher for the CP Cu when compared to the CP Al. With regard to the processed bimetallic laminates, the calculated coefficients were lower than for the CP Cu, except for the 15 mm heat-treated laminate, for which they were identical. When comparing the bimetallic laminates to the CP Al, the coefficient was higher for the laminates considering the conductors with a 15 mm diameter. However, for the 10 mm conductors, the values of the coefficients calculated for the bimetallic laminates were identical to those acquired for the CP Al conductor. The results show that, with regard to the characteristics of AC transfer, the 10 mm bimetallic laminate exhibits a more favorable behavior than the 15 mm one, regardless of the structure state.

## 4. Conclusions

Al + Cu bimetallic laminates of an innovative stacking sequence were subjected to room-temperature rotary swaging to achieve the final laminate diameters of 15 mm and 10 mm and combined with a heat treatment at 350 °C for 15 min to optimize the manufacturing conditions from the viewpoints of structure stability and electric and mechanical properties. The results show that neither the imposed swaging degree nor the subsequent heat treatment significantly affected the structure of the Al matrix, the grains of which were refined to the available minimum (−2.9 µm) after swaging to 15 mm, while the swaging degree substantially influenced the structures of the Cu components and their development during the post-process heat treatment. The behavior during DC transfer was the most favorable for the 15 mm heat-treated laminate featuring an average Cu grain size of 8.9 µm and comparable fractions of *Cube* (recrystallization) and *B* (shear deformation) texture orientations. However, the observed power loss during AC transfer was the lowest for the 10 mm bimetallic laminates, regardless of the structure state. Suffice to say, room-temperature rotary swaging was successfully used to manufacture the designed Al + Cu bimetallic laminates, and by optimizing the processing parameters of thermomechanical treatment, the properties of the final laminated conductors can be tailored for prospective DC or AC transfer (according to the requirements).

## Figures and Tables

**Table 1:** Grain size characteristics of original metals and components of processed bimetallic laminates (RS, rotary swaged, HT, heat-treated).

Component of Bimetallic Laminate	Avg. Grain Size	Min. Grain Size	Max. Grain Size	Std. Deviation
	(µm)	(µm)	(µm)	(-)
original Cu	37.2	1.3	347.4	42.0
15 mm RS Cu	11.7	3.4	89.1	9.1
15 mm HT Cu	8.9	2.3	71.2	7.7
10 mm RS Cu	6.6	2.2	42.8	5.2
10 mm HT Cu	10.2	2.3	73.0	8.8
original Al	64.1	4.4	421.6	37.1
15 mm RS Al	2.9	1.4	26.1	2.2
15 mm HT Al	2.9	1.8	9.4	1.1
10 mm RS Al	2.7	1.3	25.8	1.9
10 mm HT Al	2.8	1.6	7.6	0.9

**Table 2:** Summary of DC and AC power loss and characteristic coefficients for conductors from original metals and bimetallic laminates.

Conductor Material	CP Al		CP Cu		Bimetallic Laminate			
	15 mm	10 mm	15 mm	10 mm	15 mm RS	10 mm RS	15 mm HT	10 mm HT
**DC power loss (W)**	1.632	3.664	0.989	2.222	1.165	2.822	1.164	2.819
**AC power loss (W)**	1.637	3.667	0.996	2.226	1.172	2.824	1.173	2.822
**Coefficient k_s_ (-)**	1.003	1.001	1.007	1.002	1.006	1.001	1.007	1.001

## Data Availability

The original data supporting the research are not publicly available but a portion of the data that is not confidential is available on request from the corresponding author.

## References

[1] Jia X., Hao K., Luo Z., Fan Z. (2022). Plastic Deformation Behavior of Metal Materials: A Review of Constitutive Models. Metals.

[2] Segal V.M. (2005). Deformation Mode and Plastic Flow in Ultra Fine Grained Metals. Mater. Sci. Eng. A.

[3] Kunčická L., Klečková Z. (2020). Structure Characteristics Affected by Material Plastic Flow in Twist Channel Angular Pressed Al/Cu Clad Composites. Materials.

[4] Král P., Dvořák J., Sklenička V., Masuda T., Tang Y., Horita Z., Kunčická L., Kuchařová K., Kvapilová M., Svobodová M. (2021). Effect of Severe Plastic Deformation on Creep Behaviour and Microstructure Changes of P92 at 923 K. Met. Mater..

[5] Mohan Agarwal K., Tyagi R.K., Chaubey V.K., Dixit A. (2019). Comparison of Different Methods of Severe Plastic Deformation for Grain Refinement. IOP Conf. Ser. Mater. Sci. Eng..

[6] Kunčická L., Macháčková A., Lavery N.P., Kocich R., Cullen J.C.T., Hlaváč L.M. (2020). Effect of Thermomechanical Processing via Rotary Swaging on Properties and Residual Stress within Tungsten Heavy Alloy. Int. J. Refract. Met. Hard Mater..

[7] Hosseinzadeh A., Radi A., Richter J., Wegener T., Sajadifar S.V., Niendorf T., Yapici G.G. (2021). Severe Plastic Deformation as a Processing Tool for Strengthening of Additive Manufactured Alloys. J. Manuf. Process..

[8] Hansen N., Huang X., Hughes D.A. (2001). Microstructural Evolution and Hardening Parameters. Mater. Sci. Eng. A.

[9] Vega M.C.V., Bolmaro R.E., Ferrante M., Sordi V.L., Kliauga A.M. (2015). The Influence of Deformation Path on Strain Characteristics of AA1050 Aluminium Processed by Equal-Channel Angular Pressing Followed by Rolling. Mater. Sci. Eng. A.

[10] Kocich R., Greger M., Macháčková A. (2010). Finite Element Investigation of Influence of Selected Factors on ECAP Process. Proceedings of the METAL 2010: 19th International Metallurgical and Materials Conference.

[11] Tóth L.S., Arruffat Massion R., Germain L., Baik S.C., Suwas S. (2004). Analysis of Texture Evolution in Equal Channel Angular Extrusion of Copper Using a New Flow Field. Acta Mater..

[12] Kunčická L., Kocich R., Drápala J., Andreyachshenko V.A. (2013). FEM Simulations and Comparison of the Ecap and ECAP-PBP Influence on Ti6Al4V Alloy’s Deformation Behaviour. Proceedings of the METAL 2013: 22nd International Metallurgical and Materials Conference.

[13] Kunčická L., Kocich R., Král P., Pohludka M., Marek M. (2016). Effect of Strain Path on Severely Deformed Aluminium. Mater. Lett..

[14] Kocich R., Kunčická L., Macháčková A. (2014). Twist Channel Multi-Angular Pressing (TCMAP) as a Method for Increasing the Efficiency of SPD. IOP Conf. Ser. Mater. Sci. Eng..

[15] Orlov D., Beygelzimer Y., Synkov S., Varyukhin V., Tsuji N., Horita Z. (2009). Plastic Flow, Structure and Mechanical Properties in Pure Al Deformed by Twist Extrusion. Mater. Sci. Eng. A.

[16] Mahdavian M.M., Ghalandari L., Reihanian M. (2013). Accumulative Roll Bonding of Multilayered Cu/Zn/Al: An Evaluation of Microstructure and Mechanical Properties. Mater. Sci. Eng. A Struct. Mater. Prop. Microstruct. Process..

[17] Král P., Staněk J., Kunčická L., Seitl F., Petrich L., Schmidt V., Beneš V., Sklenička V. (2019). Microstructure Changes in HPT-Processed Copper Occurring at Room Temperature. Mater. Charact..

[18] Jamili A.M., Zarei-Hanzaki A., Abedi H.R., Mosayebi M., Kocich R., Kunčická L. (2019). Development of Fresh and Fully Recrystallized Microstructures through Friction Stir Processing of a Rare Earth Bearing Magnesium Alloy. Mater. Sci. Eng. A.

[19] Xu W.F.F., Liu J.H.H., Chen D.L.L. (2011). Material Flow and Core/Multi-Shell Structures in a Friction Stir Welded Aluminum Alloy with Embedded Copper Markers. J. Alloys Compd..

[20] Segal V. (2018). Review: Modes and Processes of Severe Plastic Deformation (SPD). Materials.

[21] Pippan R., Scheriau S., Hohenwarter A., Hafok M. (2008). Advantages and Limitations of HPT: A Review. Mater. Sci. Forum.

[22] Kunčická L., Kocich R., Strunz P., Macháčková A. (2018). Texture and Residual Stress within Rotary Swaged Cu/Al Clad Composites. Mater. Lett..

[23] Herrmann M., Schenck C., Kuhfuss B. (2016). Dry Rotary Swaging with Structured Tools. Procedia CIRP.

[24] Rogachev S.O., Sundeev R.V., Andreev V.A., Andreev N.V., Ten D.V., Nikolaev E.V., Tabachkova N.Y., Khatkevich V.M. (2022). Structure, Mechanical and Physical Properties of Cu/Al–10% La Composite Produced by Rotary Forging. Metals.

[25] Chen X., Liu C., Jiang S., Chen Z., Wan Y. (2022). Fabrication of Nanocrystalline High-Strength Magnesium−Lithium Alloy by Rotary Swaging. Adv. Eng. Mater..

[26] MacAskill I.A., LaDepha A.D.P., Milligan J.H., Fulton J.J., Bishop D.P. (2009). Effects of Cold and Hot Densification on the Mechanical Properties of a 7XXX Series Powder Metallurgy Alloy. Powder Metall..

[27] Wang Z., Chen J., Besnard C., Kunčická L., Kocich R., Korsunsky A.M. (2021). In Situ Neutron Diffraction Investigation of Texture-Dependent Shape Memory Effect in a near Equiatomic NiTi Alloy. Acta Mater..

[28] Svoboda J., Kunčická L., Luptáková N., Weiser A., Dymáček P. (2020). Fundamental Improvement of Creep Resistance of New-Generation Nano-Oxide Strengthened Alloys via Hot Rotary Swaging Consolidation. Materials.

[29] Kral P., Blum W., Dvorak J., Yurchenko N., Stepanov N., Zherebtsov S., Kunčická L., Kvapilova M., Sklenicka V. (2020). Creep Behavior of an AlTiVNbZr0.25 High Entropy Alloy at 1073 K. Mater. Sci. Eng. A.

[30] Huang A.H., Wang Y.F., Wang M.S., Song L.Y., Li Y.S., Gao L., Huang C.X., Zhu Y.T. (2019). Optimizing the Strength, Ductility and Electrical Conductivity of a Cu-Cr-Zr Alloy by Rotary Swaging and Aging Treatment. Mater. Sci. Eng. A.

[31] Kral P., Dvorak J., Sklenicka V., Horita Z., Takizawa Y., Tang Y., Kunčická L., Kvapilova M., Ohankova M. (2021). Influence of High Pressure Sliding and Rotary Swaging on Creep Behavior of P92 Steel at 500 °C. Metals.

[32] Kunčická L., Macháčková A., Petrmichl R., Klečková Z., Marek M. (2020). Optimizing Induction Heating of WNiCo Billets Processed via Intensive Plastic Deformation. Appl. Sci..

[33] Kunčická L., Kocich R. (2022). Effect of Activated Slip Systems on Dynamic Recrystallization during Rotary Swaging of Electro-Conductive Al-Cu Composites. Mater. Lett..

[34] Kocich R., Kunčická L., Dohnalík D., Macháčková A., Šofer M. (2016). Cold Rotary Swaging of a Tungsten Heavy Alloy: Numerical and Experimental Investigations. Int. J. Refract. Met. Hard Mater..

[35] Li X., Zu G., Wang P. (2013). Effect of Strain Rate on Tensile Performance of Al/Cu/Al Laminated Composites Produced by Asymmetrical Roll Bonding. Mater. Sci. Eng. A.

[36] Ma M., Huo P., Liu W.C., Wang G.J., Wang D.M. (2015). Microstructure and Mechanical Properties of Al/Ti/Al Laminated Composites Prepared by Roll Bonding. Mater. Sci. Eng. A.

[37] Lee J., Park J., Jeong H. (2018). Effect of Strain on Mechanical and Microstructural Properties of Al/Cu Claddings during Caliber-Rolling. Mater. Lett..

[38] Yang X., Li W., Xu Y., Wen Q., Feng W., Wang Y. (2019). Effect of Welding Speed on Microstructures and Mechanical Properties of Al/Cu Bimetal Composite Tubes by a Novel Friction-Based Welding Process. Weld. World.

[39] Zheng H., Zhang R., Xu Q., Kong X., Sun W., Fu Y., Wu M., Liu K. (2023). Fabrication of Cu/Al/Cu Laminated Composites Reinforced with Graphene by Hot Pressing and Evaluation of Their Electrical Conductivity. Materials.

[40] Kunčická L., Macháčková A., Krátká L., Kocich R. (2019). Analysis of Deformation Behaviour and Residual Stress in Rotary Swaged Cu/Al Clad Composite Wires. Materials.

[41] Lohit R.B., Bhovi P.M. (2017). Development of Ni-WC Composite Clad Using Microwave Energy. Mater. Today Proc..

[42] Liu B.X.X., Huang L.J.J., Geng L., Wang B., Liu C., Zhang W.C.C. (2014). Fabrication and Superior Ductility of Laminated Ti–TiBw/Ti Composites by Diffusion Welding. J. Alloys Compd..

[43] Wang C., Han J., Zhang C., Su Y., Zhang S., Zhang Z., Wang T. (2023). Temperature Dependence of Interface Characterization and Mechanical Properties of TA1/Q235 Composite Fabricated by Explosive Welding. Adv. Eng. Mater..

[44] Won D.H., Park W.S., Yi J.-H., Han S.-H., Han T.H. (2014). Effect of Welding Heat on Precast Steel Composite Hollow Columns. Struct. Concr..

[45] Didi M., Mitschang P. (2017). Induction Welding of Metal/Composite Hybrid Structures. Joining of Polymer-Metal Hybrid Structures.

[46] Cui X., Tian L., Wang D.S., Dong J.P. (2021). Summary of Thermosetting Composite Material Welding. J. Phys. Conf. Ser..

[47] Keller C., Moisy F., Nguyen N., Eve S., Dashti A., Vieille B., Guillet A., Sauvage X., Hug E. (2021). Microstructure and Mechanical Properties Characterization of Architectured Copper Aluminum Composites Manufactured by Cold-Drawing. Mater. Charact..

[48] Kim I.-K., Hong S.I. (2014). Mechanochemical Joining in Cold Roll-Cladding of Tri-Layered Cu/Al/Cu Composite and the Interface Cracking Behavior. Mater. Des..

[49] Kocich R., Kunčická L., Macháčková A., Šofer M. (2017). Improvement of Mechanical and Electrical Properties of Rotary Swaged Al-Cu Clad Composites. Mater. Des..

[50] Li T., Wang Y., Shi H., Xi L., Xue D. (2022). Impact of Skin Effect on Permeability of Permalloy Films. J. Magn. Magn. Mater..

[51] Grimm T.J., Mears L.M. (2022). Skin Effects in Electrically Assisted Manufacturing. Manuf. Lett..

[52] Kunčická L., Jambor M., Král P. (2023). High Pressure Torsion of Copper; Effect of Processing Temperature on Structural Features, Microhardness and Electric Conductivity. Materials.

[53] Verlinden B., Driver J., Samajdar I., Doherty R.D. (2007). Thermo-Mechanical Processing of Metallic Materials.

[54] Humphreys F.J., Hetherly M., Rollett A., Rohrer G.S. (2004). Recrystallization and Related Annealing Phenomena.

[55] Dalla Torre F.H., Pereloma E.V., Davies C.H.J. (2006). Strain Hardening Behaviour and Deformation Kinetics of Cu Deformed by Equal Channel Angular Extrusion from 1 to 16 Passes. Acta Mater..

[56] Russell A., Lee K.L. (2005). Structure-Property Relations in Nonferrous Metals.

[57] Liao X.Z., Zhao Y.H., Srinivasan S.G., Zhu Y.T., Valiev R.Z., Gunderov D.V. (2004). Deformation Twinning in Nanocrystalline Copper at Room Temperature and Low Strain Rate. Appl. Phys. Lett..

[58] Picu R.C., Li R., Xu Z. (2009). Strain Rate Sensitivity of Thermally Activated Dislocation Motion across Fields of Obstacles of Different Kind. Mater. Sci. Eng. A.

[59] Murashkin M.Y., Sabirov I., Sauvage X., Valiev R.Z., Sabirov I., Sauvage X., Valiev R.Z., Sabirov I., Sauvage X., Valiev R.Z. (2016). Nanostructured Al and Cu Alloys with Superior Strength and Electrical Conductivity. J. Mater. Sci..

